# Evaluating the Safety of West Nile Virus Immunity During Congenital Zika Virus Infection in Mice

**DOI:** 10.3389/fimmu.2021.686411

**Published:** 2021-06-18

**Authors:** Joshua A. Acklin, Javier D. Cattle, Arianna S. Moss, Julia A. Brown, Gregory A. Foster, David Krysztof, Susan L. Stramer, Jean K. Lim

**Affiliations:** ^1^ Department of Microbiology, Icahn School of Medicine at Mount Sinai, New York, NY, United States; ^2^ Graduate School of Biomedical Sciences, Icahn School of Medicine at Mount Sinai, New York, NY, United States; ^3^ Department of Epidemiology, Mailman School of Public Health, Columbia University, New York, NY, United States; ^4^ Scientific Affairs, American Red Cross, Gaithersburg, MD, United States

**Keywords:** flavivirus, congenital Zika syndrome, antibody dependent enhancement, vaccines, placenta

## Abstract

Antibody-dependent enhancement (ADE) is a phenomenon that occurs when cross-reactive antibodies generated from a previous flaviviral infection increase the pathogenesis of a related virus. Zika virus (ZIKV) is the most recent flavivirus introduced to the Western Hemisphere and has become a significant public health threat due to the unanticipated impact on the developing fetus. West Nile virus (WNV) is the primary flavivirus that circulates in North America, and we and others have shown that antibodies against WNV are cross-reactive to ZIKV. Thus, there is concern that WNV immunity could increase the risk of severe ZIKV infection, particularly during pregnancy. In this study, we examined the extent to which WNV antibodies could impact ZIKV pathogenesis in a murine pregnancy model. To test this, we passively transferred WNV antibodies into pregnant *Stat2^-/-^* mice on E6.5 prior to infection with ZIKV. Evaluation of pregnant dams showed weight loss following ZIKV infection; however, no differences in maternal weights or viral loads in the maternal brain, spleen, or spinal cord were observed in the presence of WNV antibodies. Resorption rates, and other fetal parameters, including fetal and placental size, were similarly unaffected. Further, the presence of WNV antibodies did not significantly alter the viral load or the inflammatory response in the placenta or the fetus in response to ZIKV. Our data suggest that pre-existing WNV immunity may not significantly impact the pathogenesis of ZIKV infection during pregnancy. Our findings are promising for the safety of implementing WNV vaccines in the continental US.

## Introduction

Antibody-dependent enhancement (ADE) is a phenomenon by which antibodies elicited from a previous infection facilitate viral entry into a susceptible cell through the engagement of Fc gamma receptors (FcɣRs) (1, 2). The most established example of ADE in humans occurs between the four serotypes of dengue virus (DENV), a mosquito-transmitted flavivirus that is estimated to cause ~390 million infections annually around the globe (3, 4). Studies show that while primary infections with DENV are typically mild, secondary infections with a heterotypic DENV serotype are more frequently severe, with increased incidence of dengue hemorrhagic fever (DHF) and dengue shock syndrome (DSS) (5, 6). ADE between the DENV serotypes is poised to occur due to the unique degree of cross-reactivity between the antigenic envelope proteins, and the fact that the four serotypes co-circulate (7–12). While high concentrations of cross-reactive antibodies may offer protection in the short term, these same antibodies can promote pathogenesis through ADE if secondary infection occurs during a time when the concentration of antibodies falls below the neutralization threshold (13, 14). Observing ADE in human populations is difficult and requires prospective studies in flavivirus endemic areas (15). In the case of dengue, while the phenomenon of severe secondary infections has been observed for decades, the correlation between antibody titers and severe disease was only directly demonstrated recently (16). A major question in the field that remains is whether this occurs with other flaviviruses in humans. In the absence of human data, *in vitro* and *in vivo* models can offer clues into this possibility. The data generated by such studies hold broad implications for the development of any flavivirus vaccine (17).

In 2016, Zika virus (ZIKV), a flavivirus closely related to DENV, received global attention after it was found to be the cause of a large outbreak that started in Brazil and spread to nearly every South and Central American country. While ZIKV has historically been associated with mild disease, this recent outbreak was much more severe (18–23). The most concerning disease outcomes were observed in pregnant women, where the virus impacted the developing fetus, resulting in spontaneous abortions and fetal abnormalities such as microcephaly (24). Based on confirmed ZIKV infections, the CDC estimates that 10% of pregnancies resulted in some form of fetal abnormality; this number increased to 15% when evaluating infections that occured during the first trimester (22, 25). Additionally, two recent studies showed that children that were exposed to ZIKV *in utero* were at greater risk of developing neurological deficits and developmental delays in their early years of life despite no overt signs of ZIKV-related damage at birth (26, 27). It remains unclear what factors may have led to the increased pathogenesis of ZIKV in the most recent outbreaks, but one possibility is the involvement of ADE. Given that antibodies against DENV can both bind to and enhance ZIKV *in vitro* and *in vivo*, and since DENV is endemic in ZIKV outbreak regions, pre-existing immunity to DENV could have been a contributing factor to the severity of the recent ZIKV outbreak (28–35). We have previously reported that passive transfer of DENV reactive antibodies can potently enhance ZIKV infection in pregnant mice. Enhanced infection resulted in heightened maternal weight loss, fetal developmental delays, damage to the developing placenta, and finally increased resorption. We further showed the dependency on antibodies as this process was maintained with purified DENV reactive IgG and reversed with FcɣR blocking (32).

In the United States, DENV does not regularly circulate as it does in South and Central America. However, West Nile virus (WNV), another closely related flavivirus, causes annual outbreaks of neurological disease, primarily meningitis, encephalitis, and acute flaccid paralysis (36). WNV is estimated to have infected at least 7 million people since its introduction into the Western Hemisphere in 1999 (37, 38). There is a great need for a WNV vaccine for human use, and should one be approved, WNV seropositivity would increase significantly. This is concerning because we and others have demonstrated that antibodies elicited by WNV are cross-reactive to ZIKV, suggesting that preexisting WNV immunity has the potential to impact ZIKV pathogenesis (31, 39, 40). In fact, during the 2016 outbreak, mosquito transmission of ZIKV occurred in both Texas and Florida, demonstrating the potential for ZIKV to expand into WNV-endemic areas (41). This risk is expected to increase as global temperatures rise (42). In this manuscript, we seek to understand the extent to which pre-existing WNV immunity impacts ZIKV outcomes during pregnancy using a *Stat2^-/-^* mouse model of ZIKV ADE.

## Materials and Methods

### Generation of Human Immune Plasma

WNV-infected blood donors were identified through screening 52,355,427 blood donations in the United States between August 2003-December 2011. Initial screening was conducted through WNV nucleic acid testing (NAT). Blood donors were identified as WNV-infected if the initial WNV NAT was reactive, repeat WNV NAT was also reactive, and WNV-specific IgM and/or IgG antibody testing results were positive on the initial blood donation or upon follow up testing. Testing for WNV antibodies was conducted by Abbott Laboratories (2003-2004; Abbott Park IL) or Focus Diagnostics (2003-2011; San Juan Capistrano CA). We received plasma from 471 WNV-infected blood donors that met these criteria. Among these, we included only those samples where positive reactivity for WNV-specific IgG on the index sample was documented (n = 146). DENV-immune plasma samples were collected as previously described (31). Random blood donor plasma samples (n = 54) that were negative for all infectious pathogens were used as negative controls. All plasma samples were individually tested for reactivity to ZIKV E protein by ELISA as previously described (31). 15 individual donors with the highest reactivity to ZIKV E protein from the DENV or WNV cohorts were pooled and used for these experiments. All control plasma failed to react to ZIKV E protein, and 15 random donors were pooled and used for the CTRL-immune plasma condition.

### Neutralization Assays

Neutralization titers were determined by co-incubating serially diluted, heat-inactivated pooled immune plasma with 10 50% tissue culture infectious doses (TCID_50_) of WNV Kunjin isolate (CH16532), DENV-1 (BC89/94), or ZIKV (PRVABC-59) in DMEM with 2% FBS. After shaking at room temperature for 1 hour, the virus-antibody mixture was added to a monolayer of Vero cells in 96 well format. Following a two-day incubation at 37°C, cells were detached from the plate using trypsin/EDTA, fixed with 4% PFA, permeabilized with PBS containing 0.2% BSA and 0.05% saponin, and stained with 4G2 antibody (1 µg/ml) for 1 hour at room temperature. After washing, cells were incubated with goat anti-mouse IgG conjugated to phycoerythrin (1µg/ml, Invitrogen) for 1 hour at room temperature. The percentage of infected cells was determined by flow cytometry using a FACS Caliber and analyzed using FlowJo2 software version 10.1.r7. The 50% inhibitory doses (IC_50_) of each plasma pool was calculated by using the four-parameter logarithmic regression in GraphPad Prism.

### *In Vitro* Antibody-Dependent Enhancement of ZIKV Infection

Antibody-dependent enhancement of ZIKV infection was measured using a flow cytometry-based assay. Briefly, serial dilutions of either non-reactive (CTRL), DENV-immune or WNV-immune human plasma were pre-incubated with ZIKV strain PRVABC-59 at an MOI of 1 for 1 hour at 37°C. Immune complexes were then co-incubated with K562 cells (5 x 10^4^) in 96 well U-bottom plates in RPMI 1640 media supplemented with 10% FBS, 2 mM L-glutamine, 10 μg/ml penicillin, and 10μg/ml streptomycin. After incubation for 48 hours at 37°C, cells were fixed with 4% PFA, permeabilized with PBS containing 0.2% BSA and 0.05% saponin, and stained with 4G2 antibody (1 µg/ml) for 1 hour at room temperature. After washing, cells were incubated with goat anti-mouse IgG conjugated to phycoerythrin (1µg/ml, Invitrogen) for 1hr at room temperature. The percentage of infected cells was determined by flow cytometry using a FACS Caliber and analyzed using FlowJo2 software version 10.1.r7. The data were then fit to a Gaussian distribution utilizing the non-linear regression functionality of GraphPad Prism, and the enhancing concentration at which 50% of maximum enhancement was reached (EC_50_) was determined utilizing the Gaussian fit.

### ZIKV Infection of *Stat2* ^-/-^ Mice

Mouse studies were carried out in an animal Biosafety Level 2+ facility under a protocol approved by the Icahn School of Medicine at Mount Sinai Animal Care and Use Committee. Breeding pairs were formed using *Stat2^-/-^* mice (provided by Christian Schindler; 8-10 week old nulliparous females and >8 week old non-virgin males). Females were then separated after plugs were detected (defined as E0.5) and infected at E6.5. Pregnant *Stat2^-/-^* mice were injected intraperitoneally with 20 µl of CTRL-, WNV- or DENV- immune plasma two hours prior to intradermal infection with 5x10^3^ PFU ZIKV (strain PRVABC-59, GenBank: KU501215.1). ZIKV was obtained originally from the CDC, passaged once in Vero cells, and sequence verified. Mice were monitored daily for weight loss. Fetal and maternal tissues were harvested at E13.5 for analysis.

### ZIKV Quantification

Total RNA was isolated from tissue homogenates using Direct-zol RNA MiniPrep Plus (Zymo Research) according to manufacturer’s protocol, and 1 μg of RNA was reverse transcribed into cDNA using the High Capacity Reverse Transcription Kit (Applied Biosciences) with random hexamer primers. The following ZIKV-specific primers (5′-TTGGTCATGATACTGCTGATTGC-3′ and 5′-CCYTCCACRAAGTCYCTATTGC-3′) were used for qRT-PCR (43), and detection of amplification was determined *via* PerfeCTa SYBR Green fast mix (Quanta) following the manufacturer’s protocol. PCR amplification was conducted in 384-well format using the Roche LightCycler 480 System. RNA quantification was determined by fitting to an *in vitro*-transcribed RNA standard.

### Cytokine and Chemokine Protein Quantification

Tissue homogenates were evaluated for 23 cytokines/chemokines by multiplexed ELISA for the following analytes: IL-17A, IL-22, IL-10, IL-2, IL-4, IFNγ, IL-21, TNFα, IL-5, IL-6, Cxcl16, Ccl4/MIP-1β, Ccl3/MIP-1α, IL-12(p70), Ccl11, IL-1β, Cxcl1/GROα, Cxcl2/GROβ, Ccl5/RANTES, Ccl7/MCP-3, Ccl2/MCP-1, Ccl12, and Cxcl10/IP-10 as previously described (44). Samples were analyzed on a Luminex MAGPIX platform. For each bead region, >50 beads were collected per analyte. The median fluorescence intensity was recorded and used to extrapolate pg/ml based on a 5P regression fitting of a standard curve of each analyte. Heat maps for median fold induction were generated in R by logarithmic base 2 transformation of the median cytokine level in the ZIKV-infected conditions divided by the median cytokine level of uninfected controls. Hierarchical clustering was performed using the hclust function in R, which stratifies based on Euclidean distance.

### Histological and Tissue Analysis

Tissues were fixed in 10% neutral-buffered formalin before embedding into paraffin and cutting into 5 μm sections. Deparaffinization and antigen retrieval was performed as previously described (31). Slides were stained with hematoxylin (Gill’s formula, Vector Laboratories H3401) and eosin Y (Sigma Aldrich E4009) according to manufacturer’s instructions. For vimentin staining, slides were blocked in Tris-buffered saline containing 0.01% Tween-20 (TBST) and 2.5% normal goat serum for 40 minutes at room temperature. After washing, slides were incubated with rabbit monoclonal anti-vimentin antibody (1:1000, clone EPR3776, Abcam) for 1 hour at 4°C, then washed and incubated with goat anti-rabbit Alexa Fluor 488 before mounting with Vectashield hard-set mounting medium with DAPI (Vector Laboratories). Full-slice images were taken with an AxioImager Z2 microscope (Zeiss) and Zen 2012 software.

*In situ* hybridization using RNAscope^®^ (ACDBio) was performed on 5 μm paraffin-embedded sections. Slides were baked at 55°C for twenty minutes for deparaffinization. Slides were then washed twice with xylene, twice in ethanol, and dried at 60°C. Slides were then incubated with hydrogen peroxide for 10 minutes at RT, and then rinsed in diH2O. Antigen retrieval was performed as per the manufacturer’s guidelines, and slides were washed with water, transferred into 100% ethanol for three minutes and air dried. Sections were treated with RNAscope Protease Plus at 40°C for 30 minutes and washed with diH2O. Fluorescence *in situ* hybridization was subsequently performed according to the manufacturer’s protocol (ACD# 323110) with RNAscope Probe V-ZIKVsph2015 (ACD #467871; binds sense RNA) as previously described (Cao et al., 2017). Slides were mounted with Vectashield hard-set mounting medium with DAPI (Vector Laboratories). Full-slice images were taken with an AxioImager Z2 microscope (Zeiss) and Zen 2012 software. Images were rendered in FIJI for final visualization.

### Quantification and Statistical Analysis

For all statistical significance indications in this manuscript, **** indicates a *p*-value ≤ 0.0001, *** indicates a *p*-value ≤ 0.001, ** indicates a *p*-value ≤ 0.01, and * indicates a *p*-value ≤ 0.05. NS indicates non significance. All sample sizes, replicate numbers, statistical tests and significance can be found in the figures and figure legends of this manuscript. Graphical representations were rendered in GraphPad Prism 8.0.1.

## Results

### *In Vitro* Characterization of WNV Antibodies Elicited Through Natural Infection

**Figure 1:**
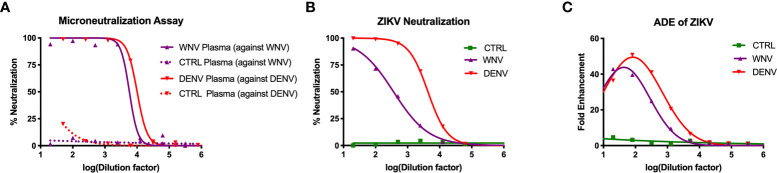
Neutralization and enhancement potential of WNV immune plasma. **(A)** The capacity of plasma from WNV-infected individuals to neutralize WNV (Kunjin isolate) was compared to the capacity of plasma from DENV-infected individuals to neutralize DENV (serotype 1) in Vero cells. Non-reactive CTRL plasma was used as a negative control for each. Neutralization is represented by the percent reduction in infection compared to virus alone. **(B)** Neutralization of ZIKV (strain PRVABC-59) by WNV, DENV, and CTRL immune plasma in Vero cells is shown. **(C)** Enhancement of ZIKV by WNV, DENV, and CTRL immune plasma was tested in K562 cells. Enhancement was determined based on percent infection over the virus alone condition. All assays were completed in technical triplicate.

To study how WNV antibodies could impact ZIKV pathogenesis, we utilized a cohort of individuals that were naturally infected with WNV identified through routine screening of the blood supply in the United States (31). To confirm antibody reactivity to WNV, we conducted a neutralization assay using the Kunjin isolate of WNV (WNV^KUN^), which is 97.7% identical at the amino acid level to the NY99 strain that was introduced to the US (45). In brief, plasma from 15 WNV-seropositive blood donors were pooled and tested for the ability to neutralize WNV^KUN^ using a flow-cytometry based microneutralization assay. Compared to plasma from geographically-matched WNV-uninfected control (CTRL) individuals, immune plasma from WNV-infected individuals showed a strong capacity to neutralize WNV (IC_50_ = 1:5,770), while the CTRL plasma showed no activity (**Figure 1A**). Given that WNV antibodies have previously been shown to cross-react with ZIKV, we next sought to assess the ability of WNV immune plasma to neutralize and enhance ZIKV (31, 40). For comparison, we used DENV immune plasma, which is known to neutralize and enhance ZIKV both *in vitro* and *in vivo* (31–34). To directly compare WNV plasma potential with DENV immune plasma, we first needed to characterize the neutralization capacity of our DENV plasma against DENV. As shown in **Figure 1A**, DENV plasma potently neutralizes DENV-1, while non-reactive CTRL plasma is incapable. Indeed, the neutralization potential of DENV plasma against DENV-1 (IC_50_ = 1:10,021) is comparable to that of WNV plasma to neutralize WNV. Therefore, any differences in the potential to neutralize and enhance ZIKV are a result of antigenic cross-reactivity, and not of differing concentrations of virus-specific antibodies. We next conducted neutralization assays against ZIKV, utilizing our CTRL, WNV, and DENV plasma. Consistent with the relative antigenic distance from ZIKV, DENV immune plasma showed ~10-fold increased capacity to neutralize ZIKV (IC_50_ = 1:4,320) compared to WNV immune plasma (**Figure 1B**) (46, 47).

Having shown the neutralizing potential of the WNV and DENV plasma against ZIKV, we next asked to what extent these samples could enhance ZIKV. WNV, DENV or CTRL plasma were serially-diluted and co-incubated with ZIKV. These virus-antibody complexes were then used to infect K562 cell, which are only susceptible to ZIKV infection through antibody-mediated entry (48). After 2 days, we evaluated infection using flow cytometry to determine fold enhancement in the presence of varying concentrations of antibodies. Enhancement potential was quantified by fitting data to a Gaussian curve, and calculating the enhancing concentration at which 50% of maximum enhancement was achieved (EC_50_). While WNV antibodies could enhance infection (EC_50_ = 390), DENV antibodies were more potent (EC_50_ = 3,470), showing ~10 fold increase in potency relative to WNV (**Figure 1C**). Together, these results demonstrate that WNV antibodies can modulate ZIKV infection *in vitro*, but to a lesser extent compared to DENV antibodies.

### Impact of WNV Antibodies on the Pathogenesis of ZIKV in Pregnant Mice

**Figure 2:**
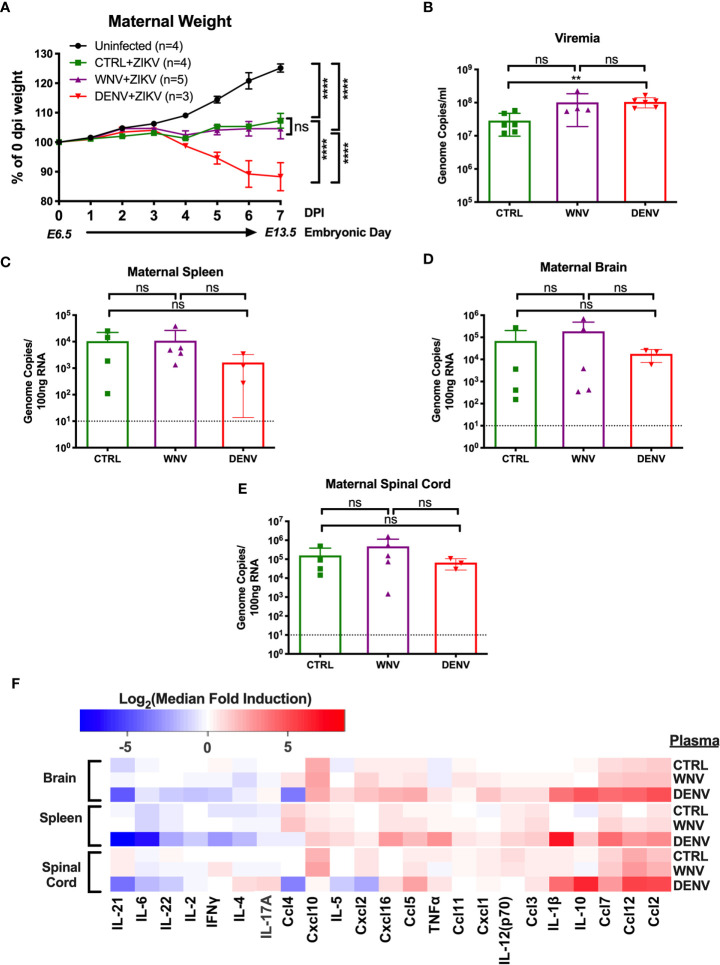
Impact of WNV antibodies on ZIKV infected dams. ZIKV-infected *Stat2^-/-^* dams that received 20μl of either CTRL (n=4), WNV (n=5) or DENV (n=3) plasma were compared to uninfected dams that also received WNV or CTRL plasma (n=4; See **Figure S1**). **(A)** Maternal weights between infection at E6.5 and tissue collection at E13.5 are shown as a percentage of starting weight at E6.5. Significance was determined by two-way ANOVA, and error bars represents SD. **(B)** Viremia was determined at 3 days post infection in ZIKV-infected dams by qRT-PCR. Mice included in this subfigure are from an unmatched cohort of CTRL (n=6), WNV (n=4), and DENV (n=6) mice. Viral loads in dams from **(A)** were determined in the maternal spleens **(C)**, brains **(D)**, and spinal cords **(E)** by qRT-PCR. For **(C–E)**, dotted lines indicate the limit of detection, and mean values with SD are depicted with the overlaid bar plots. Significance was determined by Mann-Whitney U-test. **(F)** Tissue homogenates from brain, spleen and spinal cords from ZIKV-infected mice in **(A)** were evaluated for inflammatory markers by multiplex ELISA. Data are shown as a log_2_ transformed median fold induction over the median pg/ml expression in the uninfected condition. ns, not significant; **p < 0.01, ****p < 0.0001.

Given that WNV antibodies can both bind and enhance ZIKV *in vitro*, we next evaluated how WNV antibodies could alter the course of ZIKV disease during pregnancy. To do this, we utilized ZIKV-infected pregnant *Stat2^-/-^* mice, a model we have previously employed to evaluate the enhancement of ZIKV infection in the presence of DENV antibodies (32). Pregnant *Stat2^-/-^* mice were infected on E6.5 with 5,000 PFU of the PRVABC-59 strain of ZIKV, a clinical strain from the 2015 outbreak in Puerto Rico. Two hours prior to infection, mice were injected intraperitoneally with either WNV-immune plasma, DENV-immune plasma or CTRL plasma. Uninfected control mice were also treated with either WNV (n = 2) or CTRL (n = 2) plasma. No differences were observed for maternal weights or any fetal parameters between the uninfected groups (**Figure S1**) and thus were combined into a single group. Maternal weights were monitored through E13.5, at which point maternal organs and uterine horns were evaluated. We found that while all ZIKV-infected mice lost weight over the course of pregnancy compared to uninfected mice, no significant differences were observed in the presence or absence of WNV antibodies (**Figure 2A**). Pregnant mice that received DENV plasma prior to infection steadily lost weight over the course of pregnancy, consistent with our previous findings (32). Evaluation of viremia revealed a significant increase in mice that received DENV plasma at 3 days post infection, but not in mice that received WNV plasma, compared to CTRL (**Figure 2B**). However, viral loads in maternal brains, spleens, and spinal cords revealed no significant differences in the presence of WNV antibodies or DENV antibodies by qRT-PCR (**Figures 2C–E**). While the presence of DENV antibodies in ZIKV-infected mice elevated the broad inflammatory response significantly, no striking differences existed between mice that received WNV antibodies or CTRL (**Figure 2F**).

### Impact of WNV Antibodies on ZIKV Induced Fetal Tissue Damage

**Figure 3:**
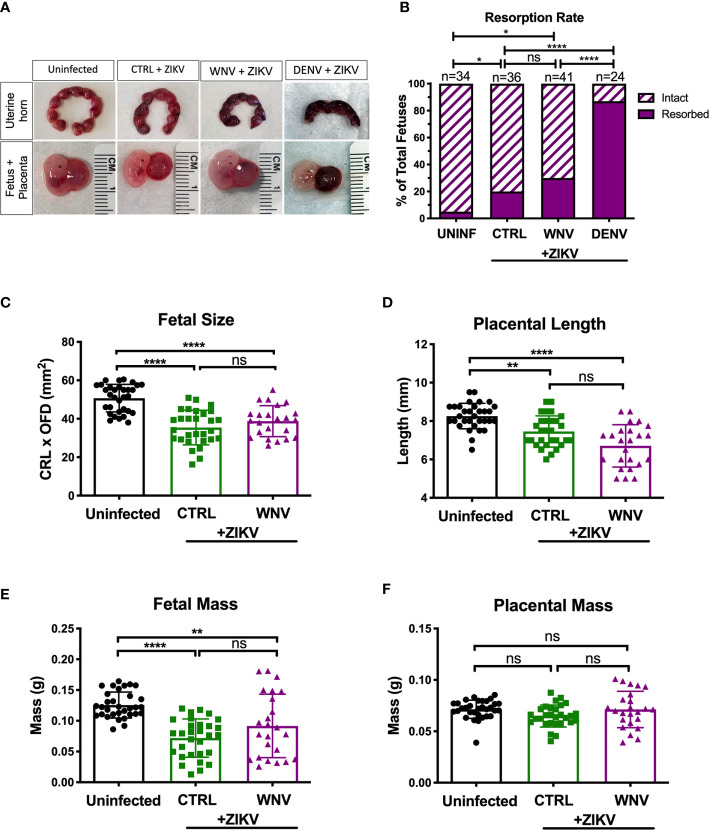
Impact of WNV antibodies on ZIKV-infected fetuses. Uterine horns were harvested and morphologically examined at E13.5 from ZIKV-infected or uninfected dams (**A**, top). Fetal and placental pairs were isolated from each harvested conceptus, and representative images are shown (**A**, bottom). The resorption rate across all pregnancies are represented by the proportion of resorbed and intact fetuses **(B)**; uninfected mice (n=34), CTRL (n=36), WNV (n=41), and DENV (n=24). Significance between groups was determined by χ^2^ analysis. Intact fetuses and placentas were measured for fetal size **(C)** by multiplying the crown-to-rump length (CRL) by the occipital-frontal diameter (OFD), placental length **(D)**, represented by diameter, fetal mass **(E)**, and placental mass **(F)**. Significance for **(C–F)** was determined by a one-way ANOVA, with a Kruskal-Wallis post-test for multiple comparisons between uninfected mice (n=32), CTRL (n=29) and WNV (n=24). Bar plot overlays for **(C–F)** depict mean and SD. Resorbed and partially resorbed fetuses were excluded from the analysis in **(C–F)**. ns, not significant; *p < 0.05, **p < 0.01, ****p < 0.0001.

We next investigated how the presence of WNV antibodies altered fetal outcomes during pregnancy in this model. We evaluated uterine horns and individual fetal-placental pairs for morphological differences upon infection to determine if the presence of WNV antibodies impacted ZIKV associated fetal damage (**Figure 3A**). Uterine horns from ZIKV infected dams, irrespective of plasma group, were darker in color and morphologically damaged compared to those from uninfected dams, with DENV plasma showing the most damage, CTRL plasma showing the least, and WNV plasma showing an intermediate phenotype. This observation was supported by quantifying the resorption rates between groups. Although the resorption rates among ZIKV-infected dams treated with WNV plasma revealed a trend towards an increase in resorption, this was not statistically significant compared to mice that received CTRL antibodies (*p* > 0.99; **Figure 3B**). This was in contrast to the mice that received DENV antibodies, where a significant increase in the resorption rate was observed (**Figure 3B**). We next evaluated fetuses and placentas for developmental defects compared to uninfected fetuses. Due to the high resorption rate, we were unable to include fetuses from dams that received the DENV antibodies in subsequent analyses and instead limited our comparisons to fetuses from pregnant dams that received WNV and CTRL antibodies. While fetuses from infected dams were significantly smaller than fetuses from uninfected dams, there were no significant differences in fetal size, fetal mass, placental length or placental mass between mice that received WNV plasma or CTRL plasma (**Figures 3C–F**).

**Figure 4:**
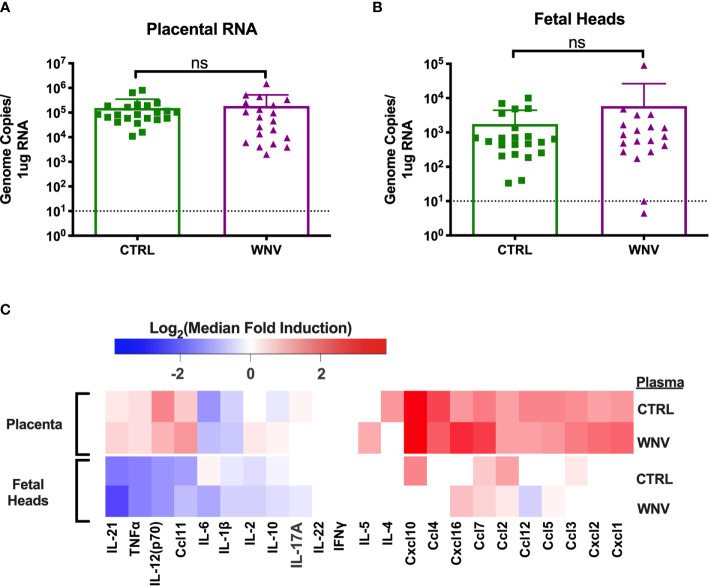
Molecular characterization of ZIKV infection in the fetus and placenta. RNA was extracted from **(A)** placentas (CTRL n=22; WNV n=20) and **(B)** fetal heads (CTRL n=22; WNV= 19) to examine viral loads by qRT-PCR. Significance was determined by Mann-Whitney U-test, and the dashed lines represent the limit of detection. Overlaid bar plots depict mean and SD. **(C)** Tissue homogenates from placentas and fetal heads were evaluated for inflammatory markers by multiplex ELISA. Data are shown as a log_2_ transformed median fold induction over the median pg/ml expression in the uninfected condition. ns, not significant.

In our previous reports, we showed that much of the damage caused by cross-reactive DENV antibodies was the result of higher viral loads in the placenta, which led to greater inflammation and placental damage (32). Therefore, we next asked whether viral replication differed in the fetal tissue in the presence of WNV antibodies by qRT-PCR. While all placentas from infected mice had viral loads in the placentas, there were no differences in viral load in mice that received WNV antibodies compared to mice that received non-specific CTRL antibodies (**Figure 4A**). Evaluation of viral loads in the fetal heads showed the same trend, with no significant differences between the two groups (**Figure 4B**).

We also asked whether the presence of WNV antibodies could alter the inflammatory landscape in the placentas and fetal heads during ZIKV infection. We examined fetal and placental homogenates from mice that received either WNV or CTRL antibodies for chemokine and cytokine production by multiplex ELISA. Examination across 21 cytokines showed no significant differences in the inflammatory milieu in either the placentas or the fetal heads obtained from ZIKV-infected pregnant dams that received WNV or CTRL antibodies (**Figure 4C**).

### Pathogenesis of ZIKV in the Placenta in the Presence of WNV Antibodies

**Figure 5:**
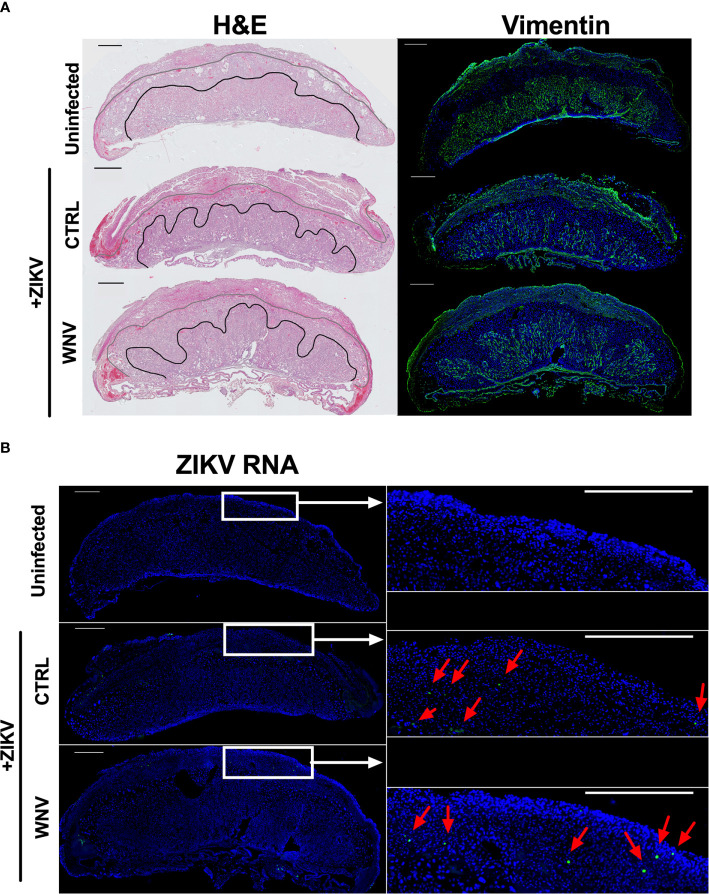
ZIKV-induced damage in the developing placenta. Placentas harvested at E13.5 from uninfected of ZIKV-infected dams that received either WNV or CTRL plasma were examined for **(A)** the disruption of the placental labyrinth zone by both hematoxylin and eosin (H&E) staining (left) and immunofluorescence staining for vimentin (right), which marks fetal endothelium (green). DAPI is shown in blue. **(B)** Placentas were evaluated for ZIKV RNA localization by *in situ* hybridization. Tiled images of representative whole placentas are shown on the left, with tiles from white boxed regions shown on the right for ease of comparison (scale = 500µm). ZIKV-infected cells (green) are highlighted with red arrows and DAPI is shown in blue.

Our previous study evaluating ADE caused by the presence of DENV antibodies implicated increased damage to the labyrinth zone of the placenta during ZIKV infection. Therefore, we wondered whether WNV antibodies had any impact on placental structure in ZIKV-infected mice. Harvested placentas across numerous pregnancies were evaluated for overall pathological changes by H&E staining (**Figure 5A**, left) as well as the density and structure of the labyrinth zone using Vimentin staining (**Figure 5A**, right). In all cases, we found a pronounced reduction in the size and density of the labyrinth zone due to ZIKV infection; however, there were no obvious differences in the placentas harvested from mice that received WNV plasma or CTRL plasma (**Figure 5A**). The extent of viral infection between mice that received CTRL or WNV antibodies was similar, consistent with the qRT-PCR results, and infection was limited to the decidual-placental interface (**Figure 5B**, red arrows) as examined by fluorescence RNA *in situ* hybridization. This is consistent with our previous findings during ZIKV infections in the absence of cross-reactive antibodies in this model (32). Together, our findings show no significant impact of WNV antibodies on the pathogenesis of ZIKV during pregnancy.

## Discussion

Our study is the first to examine the potential for WNV antibodies to enhance ZIKV infection in the context of pregnancy. We found no significant evidence that WNV antibodies mediate enhancement of ZIKV in pregnant mice. In the absence of thorough prospective human studies on the influence of WNV immunity on ZIKV infections, we hope that these data provide the first insights into the safety of introducing greater WNV immunity into ZIKV endemic regions and regions at risk of ZIKV expansion. This finding has major implications for WNV vaccine development and begins to address larger questions about the process of ADE.

ADE is known to be a function of antigenic distance, with a ‘sweet spot’ level of antigenic relatedness resulting in the possibility of enhancement. In general, higher relatedness will result in greater antigenic affinity and neutralization, while lower relatedness will result in less binding and weak or no enhancement potential. Based on ADE observed between the DENV serotypes, ~68% identity at the amino acid level for the E proteins appears to be sufficient for enhancement to be observed (49). ZIKV and the four serotypes of DENV are on average 55% similar at the antigenic E level, and *in vitro/in vivo* data suggest that enhancement *can* occur between the two viruses (50). Consistent with this notion, a recently published study showed that antibodies against ZIKV could enhance infection in children subsequently infected with DENV (51–53). However, studies that have evaluated the impact of DENV immunity on ZIKV pathogenesis in humans have largely shown that DENV immunity is protective, with one study showing a concentration dependent loss of protection against ZIKV at intermediate DENV antibody levels (54). It is possible that these seemingly conflicting data, where enhancement occurs in one direction but not the other, are the result of a wider window of severe symptomology for DENV infections compared to ZIKV infections. Alternatively, perhaps the order in which infection occurs generates antibodies against antigenic epitopes that are more or less potently neutralizing or enhancing. WNV is slightly more antigenically distant from ZIKV (~54% amino acid identity to the E protein) (49). Our study showed that WNV antibodies could enhance ZIKV at the *in vitro* level, but not at the *in vivo* level in ZIKV-infected pregnant mice. Given that the mouse model is a very sensitive model for detecting ADE, our results suggest that WNV is unlikely to alter ZIKV pathogenesis in humans, at least in the context of pregnancy. However, carefully designed prospective human studies need to test this hypothesis considering that the antigenic relatedness of these two viruses suggests that ADE could be possible. Further, the role of ZIKV specific antibodies impacting the pathogenesis of WNV has yet to be examined.

Our data also provide further evidence for how enhancement might influence ZIKV pathogenesis in pregnancy. In our report on DENV enhancement of ZIKV in this model, we identified that the placenta was the primary target for enhanced ZIKV damage (32). We found that enhancing antibodies expanded the tropism of ZIKV from the decidual-placental interface into the cytotrophoblast layer, which correlated with an increase in cytokine production, as well as in the disruption of the placental labyrinth zone. The labyrinth zone is a bed of fetal derived endothelium essential for maternal-fetal blood and nutrient transfer, and thus we hypothesized that the disruption of this zone was responsible for the fetal abnormalities noted (55). In this work, we did not find any evidence of enhancement by increased pathogenesis, inflammation or viral load. Infection was limited to the decidual interface, demonstrating cross-reactive antibodies which are not capable of increasing the infectious range of ZIKV in the placenta. It is not clear if ZIKV gains access to this compartment through direct Fc-mediated endocytosis, or if this is the result of increased access to the compartment during inflammatory responses. However, we believe that based on our data, infection of this zone is critical for the ZIKV mediated pathology observed in enhanced infections.

Our study has several limitations. One major limitation is that we only assessed a single concentration of WNV antibodies in this study (20μl/mouse). This concentration was chosen because it is the concentration where DENV antibodies can potently enhance ZIKV. However, as we showed that the enhancement potential for DENV antibodies for ZIKV is ~1 log greater than that of WNV antibodies *in vitro* (**Figure 1C**), it is possible that WNV antibodies would require ~10-fold higher concentration to achieve enhancement. Our previous data suggest that increasing the antibody concentration 10-fold would also increase mortality non-specifically due to the non-physiological concentration of circulating antibodies (31). One possibility for why DENV plasma enhances while WNV plasma does not in our experiments is because the concentration of WNV-specific antibodies is lower in the pooled WNV plasma than the concentration of DENV-specific antibodies in the DENV plasma. To address this, we quantified the number of WNV specific IC_50_’s we injected per mouse of WNV plasma and compared that to the number of DENV specific IC_50_’s injected per mouse of DENV plasma. We found that we were using a comparable concentration of IC_50_’s (<2-fold difference). Therefore, we find it unlikely that the differences observed between the WNV and DENV groups in our mouse studies are a result of differing amounts of WNV and DENV specific IgG. Instead, these data suggest that the differences we have observed are due to the relative antigenic cross-reactivity to ZIKV.

An additional limitation of this study is the use of *Stat2^-/-^* mice. ZIKV NS5 is known to bind and degrade human STAT2, but not mouse Stat2, resulting in a species barrier (56). To overcome this, the human STAT2 knock-in mouse model has recently been described for ZIKV infection in non-pregnant adult mice. Our future work will aim to utilize the human *STAT2* knock-in model for studying ADE in the context of pregnancy (34), which may be more translatable to human disease.

Ultimately, the goal of studying ADE should be to model the relative risk that immunity to any one virus poses to the severity of another. Understanding this would provide risk assessments for severe outbreak zones, vaccine development, and identification of patients at risk for severe disease sequelae. As one could imagine, this model will likely be complex and multifactorial – with antibody concentration already having been identified to be a major risk factor for ADE. Antigenic distance, neutralization capacity, antibody durability, antibody subclass, infection order and many other factors are still under investigation for their role in predisposing enhanced infection (57). This current study has begun to address the role of cross reactivity, and we hope that our findings will be helpful in building this risk model.

## Data Availability Statement

The raw data supporting the conclusions of this article will be made available by the authors, without undue reservation.

## Ethics Statement

The animal study was reviewed and approved by Icahn School of Medicine at Mount Sinai Animal Care and Use Committee.

## Author Contributions

JA conceptualized the project, conducted the experiments, data analysis, drafting and editing of the manuscript. JC conducted experiments and edited the manuscript. AM conducted experiments and edited the manuscript. JB conceptualized the project and conducted experiments. GF, DK, and SS contributed critical reagents and edited the manuscript. JL conceptualized the project, managed the project and edited the manuscript. All authors contributed to the article and approved the submitted version.

## Funding

This work was funded by NIAID grant R01AI150837 and NHLBI F31HL149295.

## Conflict of Interest

The authors declare that the research was conducted in the absence of any commercial or financial relationships that could be construed as a potential conflict of interest.
