# Supplementary material for: Evaluating the Safety of West Nile Virus Immunity During Congenital Zika Virus Infection in Mice
Source: Front Immunol. 2021 Jun 18;12:686411. doi: 10.3389/fimmu.2021.686411 (PMC8250419; doi:10.3389/fimmu.2021.686411)
Supplement: Supplementary file 1 [file DataSheet_1.docx]

Supplementary Material


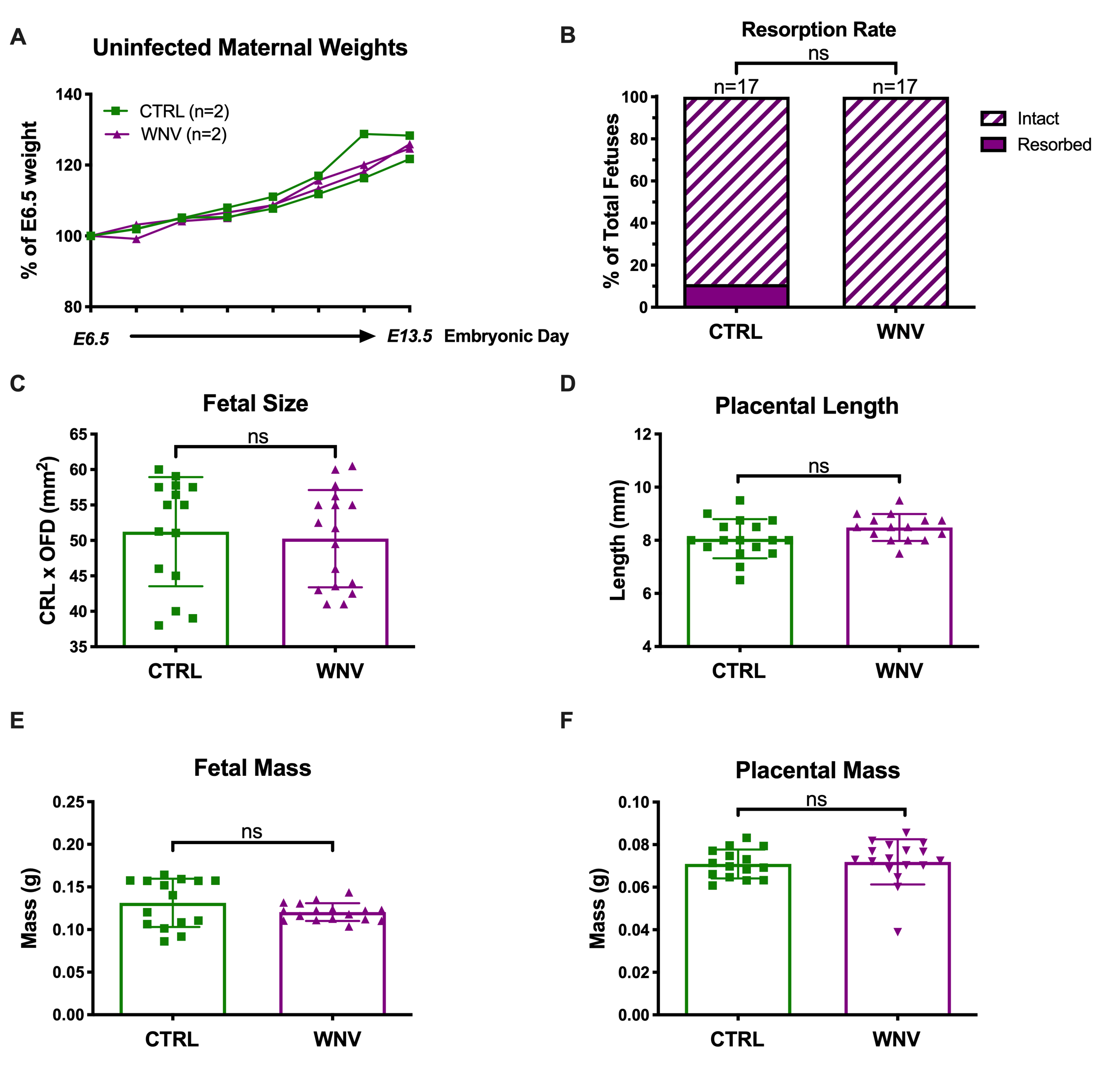


**Fig S1. Comparison of uninfected dams receiving CTRL or WNV plasma.**

Uninfected pregnant mice from Figure 1 (n=4) were given either CTRL (n=2) or WNV plasma (n=2) at E6.5, to determine if WNV plasma significantly impacts pregnancy in the absence of infection. **(A)** Maternal weights were recorded for 7 days after plasma injection. **(B)** Resorption rates between mice that received CTRL or WNV plasma are shown. Significance was determined by Fisher’s-exact test. The following fetal parameters were also compared: fetal size **(C),** placental length **(D)**, fetal mass **(E),** and placental mass **(F)**. Significance for **(C-F)** was determined by unpaired Student’s *t*-test. ns indicates non-significance.
